# Knee-specific bone quality assessment for cementless total knee arthroplasty candidate selection: a narrative review

**DOI:** 10.1186/s43019-026-00331-7

**Published:** 2026-07-10

**Authors:** Dong Hwan Lee, Dai-Soon Kwak, Sheen-Woo Lee, Sung-Min Kim, Yong Deok Kim, In Jun Koh

**Affiliations:** 1 Department of Orthopaedic Surgery, Yeouido St. Mary’s Hospital, Seoul, 07345 Republic of Korea; 2 Department of Orthopaedic Surgery, College of Medicine, The Catholic University of Korea, Seoul, 06591 Republic of Korea; 3 Department of Anatomy, Catholic Institute for Applied Anatomy, College of Medicine, The Catholic University of Korea, Seoul, 06591 Republic of Korea; 4 Department of Radiology, Eunpyeong St. Mary’s Hospital, College of Medicine, The Catholic University of Korea, Seoul, 03312 Republic of Korea; 5 Joint Replacement Center, Eunpyeong St. Mary’s Hospital, Seoul, 03312 Republic of Korea

**Keywords:** Arthroplasty, replacement, knee, Bone density, Peripheral bone quality, Tomography, X-ray computed, Cementless, Monitoring, intraoperative

## Abstract

Cementless total knee arthroplasty (TKA) has regained attention in younger, active, and obese patients, supported by advances in implant design, porous coating, and 3D printing technologies. However, the success of cementless fixation depends on adequate initial mechanical stability and subsequent osseointegration, making the assessment of bone quality at the implant–bone interface essential. Although central dual-energy X-ray absorptiometry (DXA) is useful for evaluating systemic bone health, it has been reported to inadequately reflect the localized peripheral bone quality of the distal femur and proximal tibia. Therefore, cementless TKA candidate selection requires direct evaluation of the bone quality around the knee joint, particularly at the bone resection site. This narrative review summarizes the preoperative and intraoperative methods available for knee-specific bone quality assessment in the context of cementless TKA selection. Various X-ray-based methods are accessible but limited by two-dimensional projection and protocol variability. Peripheral DXA allows localized bone mineral density measurement but lacks standardization and remains two-dimensional. Magnetic resonance imaging (MRI) has potential for assessing marrow composition and trabecular microarchitecture, but evidence supporting its use in cementless TKA selection remains limited. By contrast, computed tomography (CT)-based assessment is considered the most practical modality, offering three-dimensional evaluation and region-specific analysis. Hounsfield unit analysis on conventional CT can be readily integrated into the routine preoperative workflow, while quantitative CT and dual-energy CT provide more quantitative evaluation through volumetric bone mineral density measurement. Intraoperative assessment has the advantage of directly evaluating the implant bed; however, conventional tactile assessment remains subjective, and visual grading systems and device-based assessments have been proposed to overcome this limitation. Nevertheless, no standardized method has yet been established, and further research is needed. CT-based assessment is the most practical current modality, and intraoperative assessment is gaining objectivity. The integration of these methods with artificial intelligence-driven multi-modal assessment is expected to enable more precise and patient-specific cementless TKA candidate selection.

## Background

**Fig. 1:**
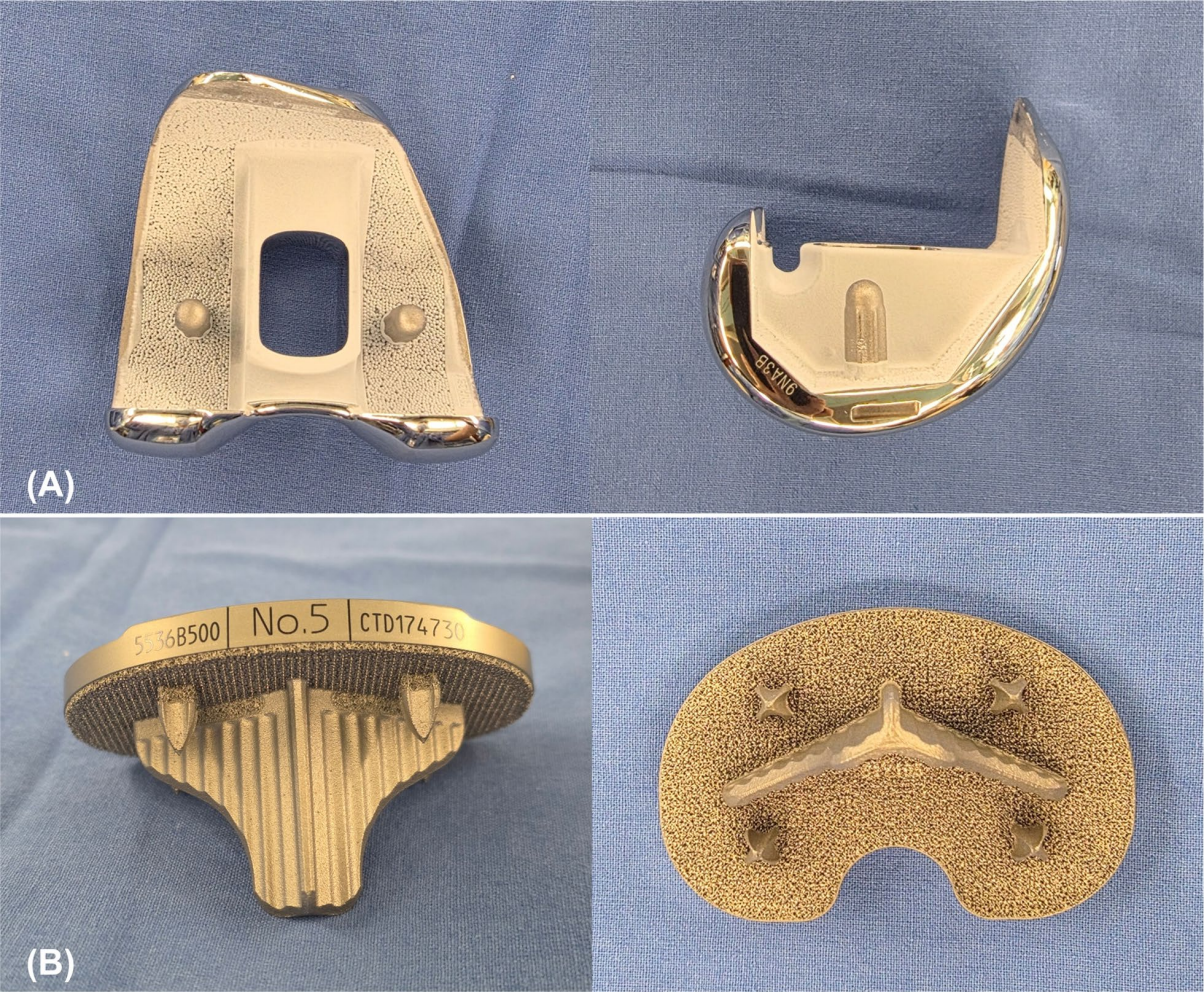
Triathlon*®* cementless total knee arthroplasty system (Stryker, Mahwah, NJ, USA), one of the currently available cementless TKA implants. **A** The femoral component is covered with hydroxyapatite (HA)-coated beads on its contact surface. **B** The tibial component features a Tritanium porous matrix produced by 3D printing, designed to mimic the trabecular structure of cancellous bone

Cemented total knee arthroplasty (TKA) has traditionally been the predominant fixation method, but the use of cementless TKA has been increasing in recent years [1]. The growing population of younger and obese patients, increasing concerns regarding aseptic loosening, and concurrent technical advances in implant manufacturing have together brought cementless implants back into the spotlight [2, 3]. The application of various technical advances to cementless implants, such as porous coating technologies and additive manufacturing through 3D printing, has raised expectations for long-term biologic fixation and a reduced risk of loosening (Fig. 1) [4, 5]. However, for these advantages to translate into clinical success, sufficient initial stability is essential. Inadequate initial mechanical stability increases the risk of implant failure, and excessive micromotion may lead to failure of bone integration [6, 7]. Therefore, ensuring initial mechanical stability is paramount, and in this regard, the assessment of bone quality is a key determinant of cementless TKA success.

## Importance of knee-specific bone quality assessment

The prevalence of osteoporosis and osteopenia is high in the TKA population, and these conditions have been reported to be underdiagnosed in the literature [8, 9]. In addition, central dual-energy X-ray absorptiometry (DXA) has been shown to inadequately reflect peripheral bone quality [10, 11]. Several studies have demonstrated that lumbar spine and femoral neck bone mineral density (BMD) have limited capacity to predict the mechanical strength of the distal femur, suggesting that determining cementless TKA suitability requires not only systemic bone health assessment but also direct bone strength evaluation of the distal femur and proximal tibia, where the implant is applied [12, 13]. Moreover, such peripheral assessment should focus not merely on bone around the knee joint in general but specifically on the bone quality surrounding the cutting surface that directly contacts the implant. In particular, in patients with knee osteoarthritis, periarticular bone density may be unevenly redistributed because of malalignment and compartment-specific loading changes [14, 15]. For example, in varus knees, the density of the medial femoral and medial tibial condyles is relatively increased, whereas the lateral compartment shows decreased density [16]. Because the initial stability of cementless implants is determined by such localized differences in bone quality, a detailed evaluation of localized bone quality is critical to the success of cementless TKA.

Therefore, this narrative review aims to summarize the current literature on knee-specific bone quality assessment, particularly from the perspective of cementless TKA candidate selection. Following the workflow required for surgical decision-making, we review preoperative and intraoperative assessments and discuss the current status and future directions of this field.

## Preoperative assessment

### X-ray-based assessment

Plain radiography is the most fundamental imaging modality in orthopedic practice and is essential in the evaluation of patients with osteoarthritis (OA). Attempts to assess bone quality using this most accessible imaging modality have continued for decades. In hip arthroplasty, indices such as the Dorr classification, cortical index, canal-to-diaphysis ratio, and canal-to-calcar ratio have long been used and have shown meaningful correlations with actual BMD [17, 18]. A recent comparative study reported that the canal-to-diaphysis ratio demonstrated the best performance among these radiographic indices [19]. Based on these findings, similar approaches have been proposed for the knee. Carlson et al. proposed the “reverse Dorr” concept, in which the distal femoral cortical ratio measured on plain knee radiographs was compared with DXA, suggesting that radiographic cortical morphology may be useful for screening osteopenia [20]. Such methods are advantageous in that they can be applied using standard radiographs alone, without additional tools or costs. However, an inherent limitation is that they do not reflect the trabecular bone strength surrounding the cutting surface, which is most relevant to cementless implant fixation. In addition, inherent constraints, such as two-dimensional projection and rotational error, further restrict its utility as a preoperative assessment tool for cementless TKA selection.

For cementless TKA candidate screening, trabecular bone quality must be assessed, and in this regard, grayscale analysis may be more appropriate. Grayscale analysis on plain radiographs quantifies pixel intensity within a predefined region of interest (ROI), and density-related surrogate values can be obtained by calibration using internal references such as an aluminum step wedge [21–23]. Because the desired ROI can be set, the trabecular bone density around the cutting surface can be measured. However, gray values obtained from routine digital radiographs are influenced by various nonbiological factors, including tube voltage, tube current–time product, detector characteristics, soft-tissue attenuation, scatter, positioning, and manufacturer-specific postprocessing algorithms. Therefore, using uncorrected gray values from standard clinical radiographs as a direct surrogate for bone quality has limited reliability.

Several studies have evaluated bone quality using grayscale and texture-based analysis on knee radiographs. Wong et al. quantified regional bone density changes in the proximal tibia after TKA using digital radiologic densitometry based on plain radiographs [24]. Grayscale values were normalized using air and implant as references, and postoperative bone loss was observed in both groups, with cementless fixation showing better bone preservation in certain regions. Although this method may be useful for tracking periprosthetic bone remodeling after arthroplasty, it has not been validated as a tool for preoperative bone quality assessment or fixation selection. More recently, Meertens et al. used a software-based densitometry platform (IBEX Bone Health) on knee digital radiographs to derive areal BMD (aBMD) and T-scores of the proximal tibia and distal femur, demonstrating high correlation with same-site DXA and excellent performance in identifying central osteoporosis [25]. These results suggest the potential for opportunistic osteoporosis screening using plain knee radiographs. However, this represents a screening tool for low bone mass, and its suitability as a fixation selection tool in TKA has not been validated.

Another potential application of plain radiography is trabecular bone texture analysis (TBTA). This approach quantifies the trabecular pattern, heterogeneity, roughness, and complexity visible on radiographs to obtain a microarchitectural surrogate [26]. In the OA literature, radiographic TBTA has been investigated as a biomarker that may reflect subchondral trabecular organization and disease progression [27]. Although the microarchitectural surrogate may partially reflect bone strength, and some studies have reported predictive value for fracture risk, its use as a bone strength prediction tool for cementless TKA selection appears limited at present [28].

In summary, owing to its accessibility, various studies have explored the assessment of bone quality using X-ray-based methods. However, the inherent limitations of two-dimensional projection, rotational error, and soft-tissue attenuation are clear. Furthermore, no validation exists for X-ray-based assessment as a preoperative screening tool for bone quality before TKA. While it may serve as a basic adjunctive tool, its application as a definitive tool for cementless TKA selection appears limited, and further research is warranted.

### Dual-energy X-ray absorptiometry (DXA)

DXA is the gold standard for the diagnosis of osteoporosis, and the T-scores of the spine and hip are essential for assessing systemic bone health. Considering the high prevalence of osteoporosis in the arthroplasty population and the inadequacy of current screening and treatment, central DXA still holds an important position in the preoperative workup [8, 9]. However, the use of central BMD (cBMD) measured by DXA for cementless TKA candidate selection has clear limitations. Although several studies have shown that cBMD correlates with peripheral BMD (pBMD), most of these studies focused on whether pBMD can be used to assess systemic bone quality, and a moderate correlation is to be expected [29, 30]. Some studies have reported that cBMD does not adequately reflect peripheral bone quality and may be inaccurate depending on medical status, age group, or specific anatomical regions [11, 31]. Because cementless TKA selection requires careful assessment of the bone quality surrounding the cutting surface, cBMD is considered insufficient as a final candidate selection tool. Suh et al. reported that distal femoral bone strength obtained through actual mechanical testing showed weak correlation with cBMD [10]. In a subsequent study by the same group, receiver operating characteristic (ROC) curve analysis demonstrated that cBMD showed poor diagnostic performance when mechanical criteria for cementless TKA suitability were applied [12]. In other words, cBMD is insufficient as a definitive tool for cementless TKA candidate selection and should be used only as a primary screening tool.

DXA around the knee joint appears somewhat more applicable to cementless TKA selection. Because the measurement region can be specified, it can better reflect local bone quality and detect regional differences. Ishii et al. measured the periarticular BMD of the femoral and tibial condyles in osteoarthritic knees and demonstrated that the load-bearing axis was closely associated with the compartmental BMD distribution [16]. In the study by Yoon et al., BMD was measured in six regions around the knee joint using knee DXA, and the correlation with cBMD differed by region [31]. Despite this potential, standardized protocols are still lacking, and further research is needed. Additionally, the limitation of two-dimensional projection means that, at present, knee joint DXA can be considered only an adjunctive evaluation tool.

### MRI-based assessment

MRI is not routinely used for bone quality assessment, but it has potential because it allows the evaluation of marrow composition and trabecular microarchitecture. However, related research is still limited, making it difficult to assess its current utility. Ehresman et al. proposed an MRI-based method for bone quality assessment in the spine [32]. The vertebral bone quality (VBQ) score, derived from type 1 relaxation time (T1)-weighted MRI, correlated well with DXA-measured cBMD. Subsequent studies have further investigated the VBQ score, and clinical applications in spine surgery are emerging [33, 34].

**Fig. 2:**
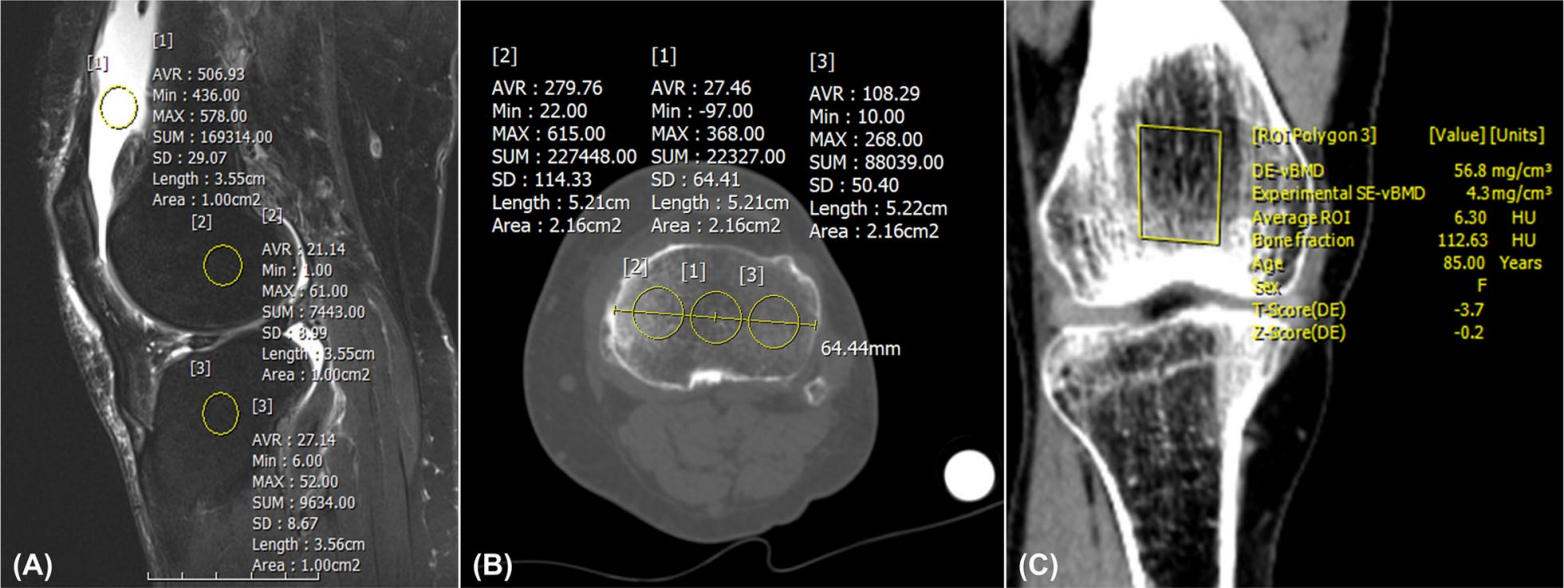
Bone density estimation using different imaging modalities. **A** MRI: based on the method described by Petrou et al.[35], the ratio between signal intensities of the femur, patella, and tibia and that of synovial fluid is calculated on fat-suppressed images. A higher ratio indicates lower bone density. **B** conventional CT-based HU analysis: the ROI can be freely positioned at the desired site. The figure illustrates the ROI placement for the proximal tibia as described by Osondu et al. [38]. **C** dual-energy computed tomography (DECT)-derived volumetric BMD (vBMD): Lee et al. [39] measured vBMD in the femoral box bone region as illustrated, and demonstrated strong correlation with actual bone strength of the corresponding box bone

Recently, a similar approach was attempted in the knee [35]. The ratio between the signal intensities of the femur, patella, and tibia on fat-suppressed images and the signal intensity of synovial fluid was measured (Fig. 2A), and the osteoporosis group showed significantly higher ratios than the normal and osteopenia groups. The femur showed the best predictive accuracy, although the predictive performance was modest at 65%. In other words, although the potential utility of MRI was confirmed, diagnostic performance still requires improvement, and with only one related study available, further validation is necessary. In addition, trabecular microarchitectural analysis using high-resolution MRI has been investigated. Using high-resolution protocols, indices analogous to subregional trabecular pattern, anisotropy, and trabecular separation can be evaluated, and associations with OA severity have been reported [36, 37]. However, the application of routine protocols is still difficult, and the relationship with bone strength has not been validated. In summary, although MRI offers clear advantages in three-dimensional evaluation and the ability to assess marrow composition and microarchitecture, the lack of established methods and insufficient validation limit its current applicability for cementless TKA selection.

### CT-based assessment

CT is currently considered the most useful modality for peripheral bone quality assessment. It provides three-dimensional structural information and allows specification of measurement regions. In addition, it is relatively inexpensive and highly accessible, as it is often acquired as part of routine preoperative workflows, particularly in robotic arthroplasty. Furthermore, considerable evidence has accumulated regarding bone quality measurement using Hounsfield units (HU) and quantitative CT (QCT) in the spine and hip, including substantial work on opportunistic CT studies [40–42]. However, given the focus on opportunistic osteoporosis screening, most of these studies have emphasized correlation with central BMD [43]. Because the present review concerns the bone quality of the knee joint undergoing cementless TKA rather than systemic bone status, we focus on knee joint specific assessment. We have organized the available CT-based assessment methods into three representative categories: conventional CT, QCT, and dual-energy CT (DECT).

#### Conventional CT

HU analysis using conventional CT has attracted attention because it is readily applicable in clinical practice without requiring specialized software or hardware. Direct selection of regions of interest is possible, and the calculation method is straightforward, which are major advantages. The HU quantifies X-ray attenuation of each voxel, standardized to water (0 HU) and air (−1000 HU), with higher values reflecting denser tissue. However, HU represents an attenuation-based estimate rather than an absolute measure of BMD and may be influenced by factors such as kilovoltage peak (kVp), scanner type, patient body habitus, and contrast administration. Inclusion of cortical bone or sclerotic areas within the ROI may further distort the results, warranting careful interpretation [44].

Studies measuring knee joint bone quality using HU on knee CT have been increasing. First, several studies have used knee CT as an opportunistic screening tool. Klinger et al. set the entire trabecular bone region as the ROI on three consecutive axial images of knee CT, measuring HU in subdivided regions of the distal femur, proximal tibia, proximal fibula, and patella [45]. When the measured HU values were compared with central DXA, good diagnostic accuracy was demonstrated in 7 of 9 regions, confirming that HU analysis on knee CT can be used as a tool for measuring bone density. Similarly, Sebro et al. performed a machine learning-based study in which volumetric segmentation of the distal femur, proximal tibia, patella, and fibula was performed on knee CT [46]. They reported that osteopenia and osteoporosis according to central DXA could be predicted using HU, with the machine learning model achieving greater predictive accuracy than CT attenuation of a single bone. These studies suggest that knee CT can serve not only as an imaging modality for alignment planning or robotic registration but also as a platform that simultaneously provides bone quality information for the knee joint.

For cementless TKA, the bone strength surrounding the cutting surface, which directly affects implant fixation, is critical. To date, few studies have directly assessed bone strength using HU analysis. One study performed CT scans of polyurethane foam blocks to obtain HU values and measured compressive strength through mechanical testing, demonstrating significant correlation [47]. More recently, in patients undergoing actual cementless TKA, mechanical testing of femoral box bone specimens was performed to measure compressive strength, which correlated with HU values measured on preoperative conventional CT in the femoral box bone region [48]. This study is significant for directly comparing HU values with bone strength. Furthermore, when mechanical criteria based on bone strength were designed for cementless TKA candidate selection, HU values demonstrated sufficient diagnostic accuracy. This indicates that HU values can be used not only for opportunistic osteoporosis screening but also as a direct protocol for cementless TKA selection. In another study, CT data from 630 patients who had previously undergone robotic-assisted cementless or cemented TKA were retrospectively analyzed, and HU values were measured in the medial, middle, and lateral areas of the proximal tibia (Fig. 2B) [38]. The mean HU of the proximal tibia in patients undergoing cementless TKA was significantly higher than that in patients undergoing cemented TKA (152.7 versus 105.6, *p* < 0.001), and younger and obese patients had higher HU values and higher rates of cementless TKA. This study also suggests that HU may be useful as a preoperative assessment tool for cementless TKA selection. The number of studies evaluating knee-specific bone strength remains limited, and few have applied HU analysis to cementless TKA selection. Further protocol standardization is therefore needed. Considering the capability for three-dimensional evaluation, direct assessment of the implant–bone interface, and accessibility, conventional CT-based assessment for cementless TKA selection is expected to be increasingly utilized in clinical practice.

#### Quantitative CT (QCT)

Although HU values from conventional CT are easy to obtain and well integrated into the routine TKA workflow, they do not provide absolute measures of bone density. QCT has the advantage of overcoming this limitation and providing absolute, quantitative measures. The conversion of HU to volumetric BMD (vBMD; mg/cm^3^) is performed using a calibration phantom of known densities, which provides a linear regression equation that is applied to the HU values within the patient’s bone ROI [49]. The vBMD obtained through this process is standardized and is currently used in clinical practice for diagnosing osteoporosis and assessing vertebral fracture risk [41, 50]. However, the addition of the calibration phantom procedure adds complexity that limits clinical implementation. To overcome this, several studies have investigated phantomless calibration methods using muscle and fat ROIs [51].

Despite this potential, studies on the use of QCT in the knee region remain limited, and no studies have evaluated bone quality specifically for cementless TKA. In one study, preoperative planning CT routinely acquired for robotic-assisted TKA was retrospectively analyzed by opportunistic QCT to measure BMD. The results showed that previously undiagnosed low BMD was relatively common [52]. That study, however, focused on osteoporosis screening rather than on knee periarticular bone quality. Another study measured subchondral BMD in the knee but focused on its correlation with OA severity [53]. To date, no studies have applied QCT to assess periarticular bone quality, bone strength, or cementless TKA candidate selection in the knee. Although future utilization is anticipated, its use may remain limited owing to the methodological complexity, and improvements in accessibility for examination and measurement are needed.

#### Dual-energy CT (DECT)

By acquiring data at two different X-ray energies, DECT performs material decomposition to separate mineral components and calculate vBMD. Although conceptually similar to QCT, DECT differs in attempting mineral characterization [54]. However, the use of DECT, similar to that of QCT, requires specialized scanners, software, and reconstruction workflows. In addition, standardized criteria for bone density assessment have not yet been established compared with DXA and QCT, and further research is needed. In the spine and hip, considerable evidence has accumulated regarding osteoporosis screening and fracture risk assessment [55, 56].

Studies on DECT-based bone quality assessment in the knee are still limited. Choi et al. measured the vBMD of the distal femur and proximal tibia and compared the results with DXA-defined osteoporosis and osteopenia, suggesting that DECT vBMD has practical value for osteoporosis screening around the knee [57]. Lee et al. directly correlated DECT vBMD with actual distal femoral bone strength using mechanical testing, demonstrating significant correlation and high diagnostic accuracy for cementless TKA candidate selection (Fig. 2C) [39]. These studies demonstrated that DECT vBMD enables localized bone density measurement with substantial accuracy and suggested its potential as a cementless TKA selection protocol.

In summary, DECT is a promising technology that enables more quantitative local bone assessment in the knee than conventional CT HU analysis. In particular, it allows three-dimensional volumetric evaluation of the periarticular bone quality of the distal femur and proximal tibia, with the possibility of opportunistic use. However, standardization is still incomplete, and the number of clinical studies is limited. The need for specialized equipment and software is also a drawback. Further studies are needed before its widespread clinical adoption for cementless TKA selection.

Conventional CT HU analysis, QCT, and DECT share major advantages in reflecting three-dimensional structures and allowing the specification of measurement regions, while each modality has slightly different characteristics with distinct advantages and disadvantages (Table 1). At present, HU analysis is most commonly used clinically because of its accessibility, while QCT and DECT are expected to develop further. Overall, CT-based assessment currently appears to be the most useful modality for cementless TKA candidate selection.

**Table 1:** Comparison of CT-based assessment modalities*

	Conventional CT (HU analysis)	Quantitative CT (QCT)	Dual-energy CT (DECT)
Principle	ROI attenuation measured on single-energy CT, expressed as HU	HU values converted to volumetric BMD (mg/cm^3^) using calibration	Material decomposition from dual-energy data, separating mineral components to calculate vBMD
Unit	HU	mg/cm^3^	mg/cm^3^
Calibration	None	Phantom or internal calibration required	Phantomless approach feasible
Advantages	Simplest and least expensive; opportunistic use possible on previously acquired knee CT	Most validated method; provides quantitative volumetric density	Direct mineral-specific quantification
Disadvantages	Not an absolute BMD; thresholds may vary by protocol	Requires additional software and complex calibration workflow	Standardization and threshold definitions incomplete; limited clinical evidence
Practicality	High—directly applicable to conventional CT	Low—high research value but burdensome for routine workflow	Emerging—promising for future application

^*^BMD, bone mineral density; CT, computed tomography; DECT, dual-energy CT; HU, Hounsfield units; QCT, quantitative CT; ROI, region of interest; vBMD, volumetric bone mineral density

## Intraoperative assessment

Bone quality assessment of the implant bed is essential for cementless TKA, and intraoperative assessment is particularly important because it enables direct evaluation of the cutting surface. However, traditional methods have relied on the surgeon’s tactile sensing, which is highly subjective. Various methods are being developed and reported to overcome this limitation. The thumb test, originally used in shoulder surgery, is a representative intraoperative assessment method in which the surgeon presses on the cutting surface with the thumb [58]. A nearly identical approach, termed the bone hardness test (BHT), has been applied to cementless unicompartmental knee arthroplasty (UKA). After tibial cutting, the surgeon presses the cut surface with the thumb and selects cemented fixation if deflection is felt or cementless fixation if not [59]. Such approaches are inherently subjective, varying with the surgeon’s tactile perception and applied force, and this limitation is difficult to overcome.

Recently, several studies have reported more systematically organized approaches. Nickel et al. systematized the haptic feedback experienced by the surgeon during bone cutting and drilling and evaluated its correlation with distal femur BMD [60]. Maniar et al. assessed bone quality intraoperatively using a visual analog scale (VAS)-like 0-to-10 scale on the basis of the surgeon’s visual and tactile evaluation and correlated the scores with femoral neck BMD measured by DXA [61]. Both studies reported strong correlations. These studies are meaningful because they have established more specific criteria and grading systems for evaluating bone quality. However, the assessment still depends on the surgeon’s perception, and these methods have not fully escaped the constraints of subjectivity.

**Fig. 3:**
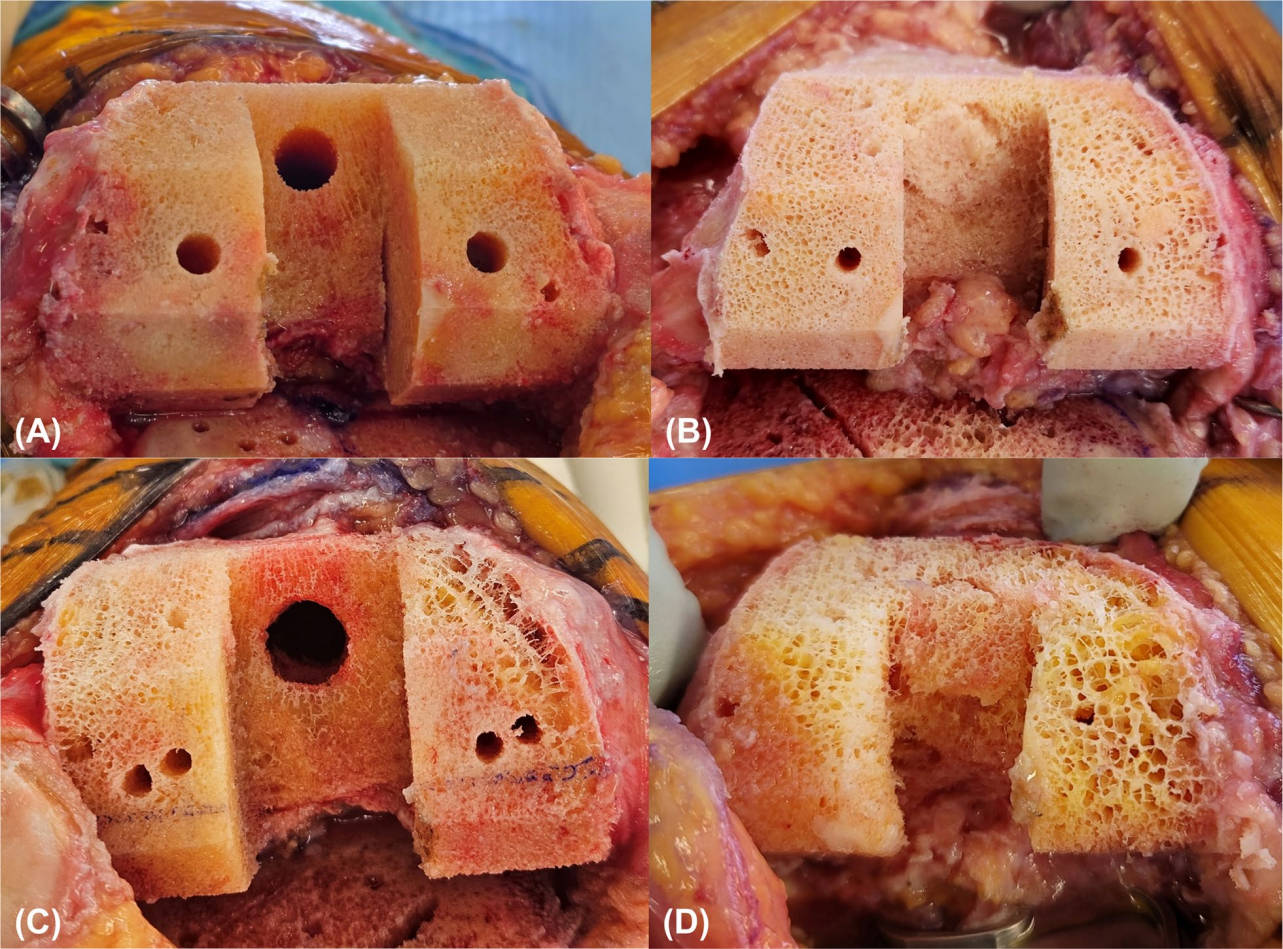
Intraoperative visual assessment system proposed by Lee et al. [62]: **A** excellent, **B** good, **C** fair, and **D** poor grade. The system classifies the femoral cutting surface into four grades on the basis of visual inspection alone, using pore characteristics and contour integrity. The visual grade demonstrated strong correlation with actual bone strength

Recent attempts have aimed to overcome these limitations of subjectivity. Lee et al. introduced a visual assessment system that classifies femoral cut surface morphology into four grades—excellent, good, fair, and poor—on the basis of visual inspection alone (Fig. 3). The grading showed strong correlation with actual distal femoral bone strength measured by mechanical testing, and the diagnostic accuracy for cementless TKA selection was excellent on ROC analysis (area under the curve (AUC) = 0.941) [62]. The advantage of this approach is its high level of objectivity, as it relies solely on visual assessment. However, the limitations include the lack of validation in tibial bone and the absence of factors beyond the visual component. Other studies have used devices to obtain more objective bone strength measurements. One study applied the Densiprobe, a wingblade coupled to a torque probe that measures trabecular peak torque, to cementless UKA selection [63]. The strength measured at the femoral peg hole correlated with BMD in various ROIs of the hip and knee on DXA. Although this method is significant in its application to cementless implant selection, it cannot be applied to instruments that do not use peg holes, and the bone strength of the peg hole region does not necessarily reflect that of the entire implant bed. Furthermore, no application has been reported in TKA.

To date, most objective intraoperative bone quality assessment methods have been validated primarily on the distal femoral cut surface, whereas evidence regarding the proximal tibial cut surface remains limited. This disparity likely reflects methodological constraints: tibial resection specimens are generally inadequate for mechanical testing, and peg holes of sufficient depth for torque-based probing are available only on the femoral side. Nonetheless, tibial component migration, subsidence, and aseptic loosening are more frequent modes of failure in cementless TKA, and tibial-side bone quality assessment is therefore essential in candidate selection. Such assessment is particularly challenging in varus osteoarthritic knees, where medial subchondral sclerosis may lead to overestimation of overall tibial implant bed quality despite the compromised bone strength of the lateral compartment and metaphyseal cancellous bone. Future studies should validate visual, tactile, and device-based assessments applied directly to the proximal tibial cut surface, correlating them with local mechanical strength and implant fixation outcomes.

Although intraoperative assessment still relies largely on the surgeon’s subjective perception, more objective indicators and studies are gradually being reported. Future advances should aim for further objectification through integration with artificial intelligence (AI) and the development of hands-on devices, with the capability to evaluate the entire implant bed. If intraoperative assessment becomes more objective and validated, it may serve as the most powerful tool for cementless implant selection because it directly evaluates the implant bed.

## Future direction

Future peripheral bone quality assessment for cementless TKA selection is expected to undergo substantial development based on AI. Machine learning has already been reported for opportunistic osteoporosis screening on knee CT [46]. Although not specific to the knee, deep learning has been used for opportunistic osteoporosis screening across various CT protocols and scanners, and another study applied deep learning to estimate BMD and assess fracture risk from radiographs [64, 65]. Because the setting of the ROI and the calculation of attenuation within that region are processes that can ultimately be automated, AI integration will likely drive further advancement. Furthermore, more accurate cementless TKA selection will likely require implant bed-specific bone quality assessment. Studies published to date have evaluated knee bone quality uniformly across the entire knee joint or in specific regions such as the box bone. As previously mentioned, because bone strength varies according to patient-specific factors such as weight-bearing and alignment, site-specific measurement of the bone bed strength where the implant will be applied is ultimately what will ensure cementless TKA success. We anticipate that bone quality and strength will be measured in separate ROIs on the basis of the implant-specific geometry of the femoral and tibial components. Beyond this, comprehensive cementless TKA suitability assessment tools may also be developed. Such tools would integrate preoperative imaging-based bone quality measurement, intraoperative assessment, cortical support, and patient factors such as weight and sex.

In addition, peripheral assessment must be integrated not only with fixation choice but also with perioperative bone health optimization. When low BMD is identified preoperatively, general bone health should be optimized through antiresorptive or anabolic therapy, vitamin D repletion, and nutritional support. As this may relate to osseointegration, it can also benefit cementless TKA success. Furthermore, optimizing bone health is essential for maintaining the patient’s overall health and quality of life and should therefore be addressed in parallel.

## Conclusions

The success of cementless TKA cannot be guaranteed by improvements in implant design or technology alone; accurate assessment of bone quality around the knee joint is essential, as it determines the achievement of initial rigid fixation. Current evidence indicates that central BMD does not correlate strongly enough with peripheral bone quality; therefore, the bone quality of the knee joint should be assessed separately. Knee joint DXA and X-ray-based methods have promising value as screening tools but are limited by their two-dimensional nature, making them suitable only for adjunctive roles, while MRI currently has insufficient evidence for routine use. Among current modalities, CT-based assessment is the most practical and powerful: it provides three-dimensional local bone evaluation, integrates well with the routine TKA workflow, and is supported by the largest body of evidence, including studies confirming good correlation with actual bone strength. Intraoperative visual-tactile assessment remains essential, and recent attempts at objectification have shown meaningful results. Further standardization and objectification, combined with CT-based assessment, could enhance the precision of cementless TKA selection.

A critical and unresolved issue in this field is the lack of clear evidence regarding the minimum bone strength required to prevent failure of cementless TKA. Based on the available literature, Lee et al. adopted 2.5 times body weight as the minimum required strength and applied this criterion to validate the diagnostic accuracy of various assessment methods for candidate selection [48, 66, 67]. In clinical practice, however, the bone strength required for stable cementless fixation is influenced by multiple interacting patient-specific factors, such as body mass index, activity level, postoperative alignment, implant position, and soft tissue balance. Therefore, additional studies that integrate these multiple factors are essential to define the minimum required bone strength for cementless TKA candidate selection. The integration of artificial intelligence, multi-modal imaging, site- and implant-specific assessment, and accurate estimation of the minimum required bone strength will be key to achieving more precise and individualized cementless TKA candidate selection.

## Data Availability

No datasets were generated or analyzed during the current study.
